# Nuclear Medicine Imaging Biomarkers in Parkinson’s Disease: Past, Present, and Future Directions

**DOI:** 10.3390/medsci13040308

**Published:** 2025-12-07

**Authors:** Anna Lisa Martini, Stelvio Sestini, Dinahlee Saturnino Guarino, Paola Feraco

**Affiliations:** 1 Unit of Nuclear Medicine, Department of Diagnostic Imaging, N.O.P.—S. Stefano, U.S.L. Toscana Centro, 59100 Prato, Italy; 2 Department of Radiology, Perelman School of Medicine, University of Pennsylvania, Philadelphia, PA 19104, USA; 3 Centre for Medical Sciences (CISMed), University of Trento, 38122 Trento, Italy

**Keywords:** Parkinson’s disease, Nuclear medicine, PET, radiotracers, neuroimaging

## Abstract

Parkinson’s disease (PD) is a multifaceted neurodegenerative disorder characterized by dopaminergic neuronal loss and widespread α-synuclein pathology. Nuclear medicine imaging offers essential in vivo tools for early diagnosis, differential assessment, and monitoring disease progression. This review summarizes key PET and SPECT radiotracers targeting dopaminergic synthesis and transport, vesicular storage, post-synaptic receptors, neuroinflammation, and protein aggregation, highlighting their roles in clinical evaluation and phenotyping. Clinically, these modalities support earlier recognition of PD, distinction from atypical parkinsonian syndromes, and assessment of non-motor involvement. Future directions include the development of selective α-synuclein tracers and multimodal imaging strategies to refine prodromal detection and guide personalized therapeutic interventions.

## 1. Introduction

Parkinson’s disease (PD) is a complex neurodegenerative disorder that poses significant diagnostic and therapeutic challenges, particularly in its early stages and in differentiating it from atypical parkinsonian syndromes. Given that PD and other parkinsonian syndromes present with overlapping clinical features yet are underpinned by distinct molecular mechanisms, employing biomolecular imaging techniques is essential for elucidating the pathophysiological processes involved. Indeed, while the clinical diagnosis remains primarily based on motor features, advances in molecular imaging have opened up new avenues for improving diagnostic accuracy, monitoring disease progression, and guiding therapeutic decisions [1,2]. Despite significant advances in understanding its pathophysiological mechanisms, early diagnosis and objective monitoring of disease progression remain major clinical challenges. In this context, molecular imaging techniques have emerged as essential tools for diagnosis, patient stratification, therapeutic monitoring, and the identification of disease-specific biomarkers. The aim of this review is to provide a comprehensive overview of molecular imaging tracers used in PD, focusing on both established and novel agents, with a focus on their roles in routine clinical practice, as well as their potential utility in research and future therapeutic stratification. We will trace the historical development of radiopharmaceuticals and explore key molecular targets, such as dopamine transporters, postsynaptic receptors, and enzymes involved in dopamine synthesis or degradation, highlighting their relevance in the diagnostic workflow and disease staging. Classic radiotracers, targeting presynaptic dopaminergic function such as dopamine transporters (DAT), vesicular monoamine transporter type 2 (VMAT2), and aromatic L-amino acid decarboxylase (AADC), have proven valuable in supporting the diagnosis of PD and distinguishing it from non-degenerative causes of parkinsonism [3,4,5]. However, their utility in monitoring disease progression or capturing non-dopaminergic pathology remains limited [6]. In recent years, the development of novel radiotracers has expanded the scope of molecular imaging beyond the dopaminergic system. These include agents targeting neuroinflammation (e.g., TSPO ligands), synaptic density (e.g., SV2A ligands), mitochondrial dysfunction, and, most notably, α-synuclein (α-Syn) aggregation, the pathological hallmark of PD [7,8,9,10]. Although still largely confined to research settings, these tracers hold promise for translational applications such as patient stratification in clinical trials, early disease detection, and evaluation of disease-modifying therapies [11]. Finally, we will discuss future directions, including the integration of molecular imaging into personalized medicine approaches, the development of tracers targeting α-Syn, considered the pathological hallmark of PD, and the role of these agents in clinical trials for disease-modifying and neuroprotective therapies.

## 2. Clinical History of Parkinson’s Disease

### Preclinical, Prodromal, and Clinical Stages of PD

**Figure 1:**
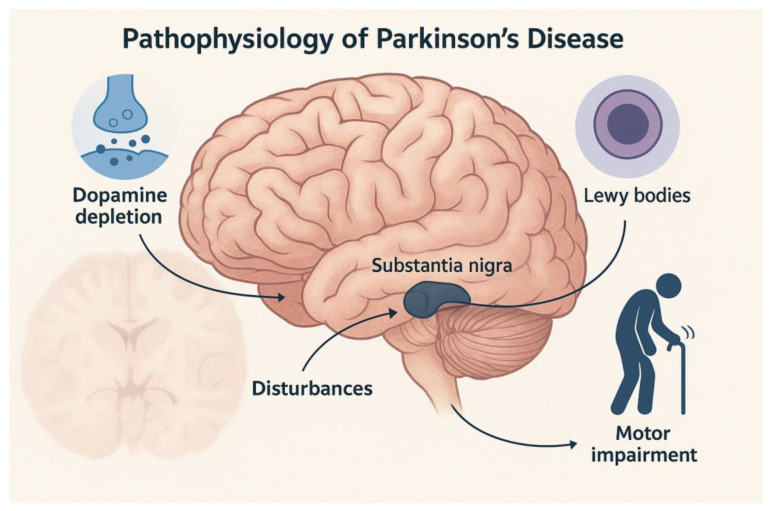
The illustration depicts the principal neurobiological mechanisms underlying PD, including dopaminergic neuron loss in the substantia nigra pars compacta, nigrostriatal dopamine depletion, and the accumulation of α-Syn aggregates forming Lewy bodies. These alterations lead to disrupted basal ganglia circuitry, reduced striatal dopamine transmission, and the emergence of the characteristic motor symptoms (bradykinesia, rigidity, tremor) and non-motor manifestations of the disease.

PD is a systemic neurodegenerative disorder characterized by widespread deposits of α-Syn in both the central and peripheral nervous systems, including the enteric nervous system [12]. Notably, significant involvement of the autonomic nervous system, both parasympathetic and sympathetic branches, as well as the entire gastrointestinal tract, is observed in both manifest PD and its prodromal phases. Autonomic symptoms involving the genitourinary and gastrointestinal systems often precede clinical diagnosis [13,14,15,16,17]. One prominent hypothesis proposes that α-Syn aggregation originates in highly branched, unmyelinated neuronal terminals and subsequently propagates via retrograde axonal transport [18,19,20]. This mechanism may explain the early and pronounced accumulation of α-Syn in autonomic nerves [2,21]. Clinical manifestations in PD depend on the specific neuronal systems affected and the sequential involvement of these systems. A definitive diagnosis of PD is typically made when classical motor symptoms, resulting from presynaptic dopaminergic system degeneration, become apparent [21,22]. Motor symptoms generally emerge after a loss of 50–70% of nigrostriatal dopamine function [2,23], implying that pathological changes in the substantia nigra begin several years before overt motor signs develop (Figure 1) [24].

During the prodromal phase, especially in individuals with the “body-first” subtype, non-motor symptoms such as constipation, reduced olfactory function, sleep disturbances, and depression may be present, albeit insufficient to fulfill diagnostic criteria. Consequently, by the time PD is clinically diagnosed, the disease has often advanced to a stage where the efficacy of disease-modifying treatments might be compromised [25,26]. Among the early non-motor signs, idiopathic REM sleep behavior disorder (iRBD), characterized by a lack of muscle atonia during REM sleep leading to enactment of dreams, has emerged as a robust predictor; longitudinal studies indicate that over 80% of individuals with iRBD eventually develop PD or dementia with Lewy bodies (DLB) [27,28,29]. Early detection of these pathological changes, even before structural alterations are evident, is crucial for implementing timely therapeutic interventions to prevent irreversible neuronal damage.

## 3. Radiotracers in Parkinson’s Disease

**Figure 2:**
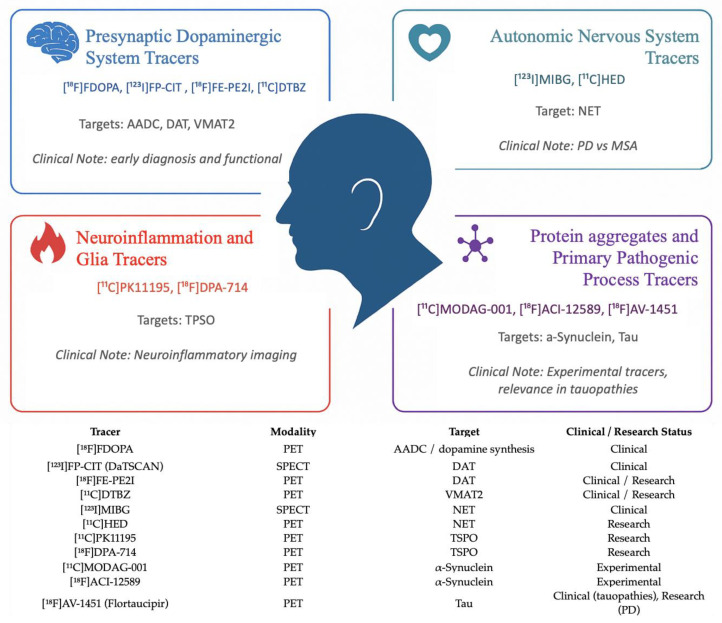
Molecular imaging tracers in PD and atypical parkinsonian syndromes, categorized by molecular target and clinical application, and embedded summary table of tracers, imaging modalities, molecular targets, and current clinical/research status. The schematic highlights representative PET and SPECT radiotracers used to assess the presynaptic dopaminergic system (AADC, DAT, VMAT2), the autonomic nervous system (NET), neuroinflammation (TSPO), and pathogenic protein aggregates (α-Syn, tau). Each category includes example tracers and summarizes their primary clinical utility, from early diagnosis and differential assessment to emerging roles in biomarker-based research. This combined graphical and tabular representation provides a unified view of established and emerging molecular imaging biomarkers relevant to the diagnosis, phenotyping, and future prognostic stratification of PD and related disorders.

Several radiotracers have been developed to evaluate distinct pathological aspects of PD and atypical parkinsonian syndromes. These radiotracers enable the noninvasive assessment of altered synaptic transmission and neurotransmitter dynamics, loss of specific neuronal structures, including autonomic nerve terminals, and accumulation of pathological proteins such as α-Syn and tau (Figure 2).

The radiotracers currently approved and used in clinical practice include:

- [^18F]FDOPA: approved for PET imaging to evaluate presynaptic dopaminergic function and assess nigrostriatal integrity [30];
- [^123I]FP-CIT (DaTSCAN): approved for SPECT imaging of the presynaptic dopaminergic system, assessing dopamine transporter (DAT) availability in the striatum [31];
- [^123I]MIBG: used in SPECT imaging to assess cardiac sympathetic innervation, useful in differentiating PD from multiple system atrophy (MSA) and progressive supranuclear palsy (PSP) [32]. These tracers represent the current standard of care in clinical molecular imaging of PD, aiding in diagnosis, differential diagnosis, and disease staging (Table 1).

In addition, several lines of research are currently focused on the development and validation of novel PET and SPECT radiotracers targeting different molecular pathways involved in PD pathophysiology, which are not yet approved for clinical use (Table 2).

## 4. The Dopaminergic System

Dysfunction within the dopaminergic system is a well-established hallmark of PD, making its various components prime targets for imaging studies. Both presynaptic and postsynaptic radiotracers are available, each interrogating different steps of the dopaminergic pathway, from dopamine synthesis and vesicular storage to transporter density and receptor availability [33]. Although DAT, VMAT2, and [^18F]FDOPA tracers are all used to assess presynaptic dopaminergic integrity, their biological targets and interpretative nuances differ substantially. DAT radiotracers primarily reflect dopamine transporter density and are sensitive to medication effects and compensatory mechanisms [31]. VMAT2 tracers quantify vesicular monoamine transporter activity, providing a more stable measure of dopaminergic terminal density, less influenced by synaptic dopamine fluctuations [34]. In contrast, [^18F]FDOPA PET quantify AADC activity and dopamine synthesis capacity but can be confounded by peripheral metabolism and variable tracer kinetics [30]. These complementary tools allow clinicians and researchers to capture distinct pathophysiological mechanisms underlying PD and atypical parkinsonian syndromes.

### 4.1. Dopamine Synthesis and Metabolism

PET using [^18F]FDOPA, a fluorinated analog of L-DOPA, enables the in vivo assessment of the enzymes involved in dopamine synthesis [30]. The uptake of [^18F]FDOPA in the striatum reflects the density of catecholaminergic nerve terminals and the activities of enzymes such as AADC and VMAT2. Conversely, the washout of the tracer is influenced by the metabolic actions of catechol-O-methyltransferase (COMT) and monoamine oxidase (MAO) [34]. In healthy individuals, striatal [^18F]FDOPA accumulation increases consistently over the first 90–120 min post-injection [35]. In PD, however, a reduction in [^18F]FDOPA uptake is observed in the striatum, typically in an asymmetric pattern with more pronounced decreases in the posterior putamen compared to anterior regions [35,36]. This reduced uptake correlates with the severity of certain motor symptoms, such as bradykinesia and rigidity [37], while non-motor features like cognitive deficits and depression appear to be driven by additional pathological mechanisms [38]. Furthermore, clinical studies have demonstrated that interventions such as dopaminergic cell transplantation can partially restore striatal [^18F]FDOPA uptake [39]. Serial measurements have also suggested that the preclinical phase of PD, during which neurodegeneration is active yet asymptomatic, may extend up to seven years [40]. Notably, [^18F]FDOPA has received regulatory approval in Europe (2006) and in the United States (2019) for differentiating PD from essential tremor and other parkinsonian syndromes [41]. Another radiotracer, 18F-fluoro-m-tyrosine ([^18F]FMT), exhibits higher affinity for AADC and produces fewer confounding radiometabolites, potentially offering a more direct measure of AADC activity within the striatum [42]. In small-scale studies, [^18F]FMT has shown a stronger correlation with certain clinical manifestations compared to [^18F]FDOPA, although neither tracer has consistently correlated with non-motor symptoms such as cognitive or affective dysfunction [42].

### 4.2. Dopamine Transporter (DAT) Activity

The DAT is an 80-kDa protein located on the presynaptic membrane of dopaminergic neurons, originally described approximately three decades ago. Structurally, it encompasses 12 transmembrane domains with both carboxyl and amino termini residing intracellularly. DAT undergoes external glycosylation and is prominently localized in the axonal membranes of nigrostriatal dopaminergic neurons [43,44]. Although its highest expression is found in the striatum and nucleus accumbens, lower levels of DAT occur in regions such as the globus pallidus, cingulate cortex, olfactory tubercle, amygdala, and midbrain [45].

By regulating dopamine reuptake from the synaptic cleft into the presynaptic neuron, DAT plays a critical role in the spatial and temporal control of dopaminergic signaling [46]. Its activity is modulated by presynaptic receptors, intracellular protein kinases, and trafficking mechanisms that affect its internalization and recycling [47,48,49]. This transporter exerts a profound influence on motor function, cognition, and the reward system [50]. Pharmacologically, it serves as a binding site for substances of abuse (e.g., cocaine and amphetamine) and for therapeutic agents such as methylphenidate and bupropion [51,52]. Striatal DAT density declines at an approximate rate of 6–7% per decade in humans [53,54,55], making it a suitable marker for assessing dopaminergic terminal innervation [56].

**Figure 3:**
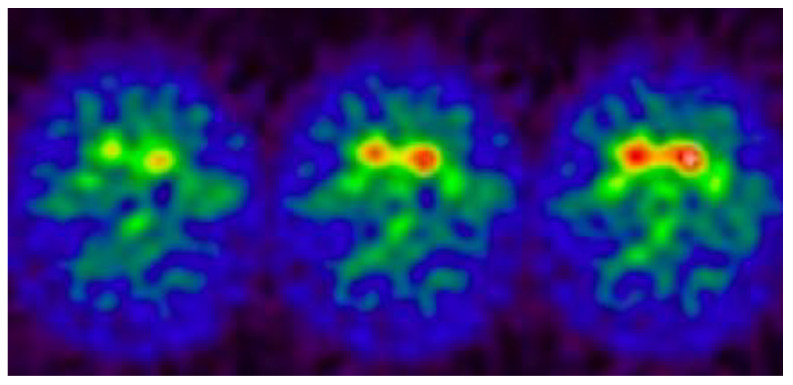
An example of a brain PET DAT-SCAN in a patient with PD, reveals bilateral low uptake in the putamen and in the head of the caudate (red uptake in the figure) with particular evidence in the right side.

In PD, DAT imaging is widely employed to evaluate the integrity of presynaptic dopaminergic neurons (Figure 3). Various DAT-targeting ligands (often derived from tropane-based structures) have been developed to diagnose PD and monitor disease progression [57,58].

Several tracers, including [^11C] cocaine, [^123I]β-CIT, [^123I]ioflupane (FP-CIT), [^11C]/[^18F]CFT, [^123I]/[^11C]PE2I, and [^11C]methylphenidate, have progressed to phase III or IV clinical trials.

Meta-analytic data indicate that DAT imaging provides sensitivities between 78–95%, specificities between 85–97%, and AUC values of 0.90–0.97 for distinguishing PD from non-degenerative or atypical parkinsonian syndromes. For FP-CIT SPECT specifically, diagnostic performance is robust, with pooled sensitivity around 90%, specificity around 92%, and AUC ≈ 0.94, while β-CIT and TRODAT-1 studies report values within a similar range [31].

Numerous SPECT and PET studies underscore the strong relationship between striatal DAT loss and PD severity, particularly in the posterior putamen [59,60,61,62]. For instance, a meta-analysis revealed a systematic decrease in dopamine levels across both the anterior and posterior putamen and in the caudate nucleus, aligning with clinical severity [60]. Additionally, 99mTc-TRODAT-1 binding has been inversely correlated with motor symptom progression [31,61]. Beyond motor impairment, DAT dysfunction is also implicated in various non-motor symptoms of PD, including RBD, autonomic disturbances, cognitive decline, and mood disorders. In individuals with RBD, approximately 58.6% exhibit decreased FP-CIT uptake, and these patients display a heightened risk of eventually developing PD or dementia with Lewy bodies [28]. Furthermore, some evidence suggests that DAT reductions in the caudate nucleus or ventral striatum may contribute to depressive symptoms and cognitive impairment [62,63,64,65,66,67,68]. However, results regarding the precise role of DAT alterations in non-motor aspects remain partially inconsistent and warrant additional investigation.

Overall, DAT imaging remains a pivotal tool for elucidating presynaptic dopaminergic integrity, enabling clinicians and researchers to track disease progression and to differentiate Parkinson’s disease from other parkinsonian syndromes. Moreover, research suggests that cognitive deficits in PD are closely associated with diminished DAT expression in the striatum. Investigations using FP-CIT imaging also indicate that reductions in caudate DAT binding correlate with olfactory impairments in PD patients [69,70].

### 4.3. Vesicular Monoamine Transporter Type 2 (VMAT2)

VMAT2 is a protein embedded in the membrane of synaptic vesicles, where dopamine is stored. Its function can be imaged using PET tracers such as ^11Cdihydrotetrabenazine ([^11C]DTBZ) and 9-[^18F]fluoropropyl-(+)-dihydrotetrabenazine ([^18F]DTBZ or [^18F]AV-133).

In PD, presynaptic degeneration produces a quantifiable reduction in striatal VMAT2 tracer uptake, with diagnostic sensitivities typically ranging from 85% to 95%, specificities between 85% and 96%, and AUC values of 0.92–0.97, depending on the tracer and analytic model [71,72]. Comparative within-subject studies using [^18F]FDOPA, [^11C]-d-threo-methylphenidate, and [^11C]DTBZ have shown that the magnitude of striatal signal reduction differs across targets: on average, [^11C]DTBZ shows a 20–30% greater decline than [^18F]FDOPA, while [^11C]-d-threo-methylphenidate demonstrates an additional 10–15% reduction beyond [^11C]DTBZ [73,74]. These quantitative differences suggest that VMAT2 availability may act as a less biased and more linearly responsive biomarker of disease progression than measures of dopamine synthesis or DAT expression.

Preclinical and clinical findings further indicate that VMAT2 binding demonstrates lower variability (<10%) in response to dopaminergic medications, compared with DAT tracers or [^18F]FDOPA (often showing medication-related variability of 20–40%) [75]. Importantly, modeling studies estimate that VMAT2 density may begin to decline 15–20 years before the onset of motor symptoms, underscoring its potential to track the pre-symptomatic phase of PD [76,77].

### 4.4. Dopamine Receptors

Imaging post-synaptic dopamine receptors provides valuable insights into the integrity of the dopaminergic system beyond the presynaptic terminals. Most dopamine receptor tracers target either the D1-like (D1 and D5) or D2-like (D2, D3, and D4) receptor families. For D1-like receptors, available radioligands are limited, with PET tracers such as [^11C]NNC112 and [^11C]SCH23390 being predominantly utilized [78]. In contrast, a variety of tracers for D2/3 receptors exist, including SPECT ligands like [^123I]IBZM and PET ligands such as [^11C]raclopride, [^18F]desmethoxyfallypride, and (+)-[^11C]PHNO [78]. These tracers are particularly effective in imaging regions with high D2/3 receptor density, such as the striatum. For extrastriatal areas, where receptor densities are lower, ultra-high-affinity tracers like [^11C]FLB457 and [^18F] fallypride are employed [79].

Quantitatively, D2/3 receptor SPECT imaging (particularly with IBZM) displays sensitivities of 75–88%, specificities of 80–92%, and AUC values of 0.85–0.92 for distinguishing PD from atypical parkinsonian syndromes. PET ligands such as [^11C]raclopride generally perform similarly or slightly better, reaching sensitivities of 85–90% and specificities of 88–95%, with AUC values of 0.90–0.95 in differential diagnostic applications [80].

An important characteristic of D2/3 tracers is their high responsiveness to intrasynaptic dopamine levels [81], with (+)-[^11C]PHNO showing the greatest sensitivity. In challenge paradigms (e.g., amphetamine), (+)-[^11C]PHNO can detect dopamine-induced occupancy changes of 10–25%, markedly higher than the 5–10% typically observed with [^11C]raclopride [82]. PET studies consistently report no significant quantitative differences in D1-like receptor availability between PD patients and healthy controls [83,84]. Conversely, D2/3 receptor binding in PD is often normal or mildly elevated during early stages but shows a progressive decline over time, particularly in extrastriatal regions, where reductions can exceed 20–30% relative to controls [85]. In atypical parkinsonian syndromes, baseline striatal D2/3 availability is significantly lower, typically showing reductions of 30–50%, making these tracers useful for differential diagnosis [86,87,88].

Receptor occupancy studies have also demonstrated quantifiable engagement of D2/3 targets by endogenous dopamine and D2/3 agonists following anti-PD treatments [89]. Given the relevance of D3 receptors for dopaminergic therapeutics [90], selective imaging with D3-preferring ligands such as (+)-[^11C]PHNO, whose binding correlates with motor impairment and mood alterations, has shown diagnostic performance with AUC values around 0.88–0.93 [91,92]. Ongoing research is focused on developing even more selective D3 PET radiotracers [93]. Despite the availability of multiple tracers, the clinical application of dopamine receptor imaging in PD remains primarily within the realm of research. given the more robust diagnostic performance of presynaptic markers.

## 5. [^123I]MIBG SPECT Imaging

Cardiac [^123I]MIBG SPECT imaging is used to assess the integrity of cardiac sympathetic innervation, which reflects the health of the peripheral autonomic nervous system [26]. [^123I]MIBG, a guanethidine analog, is taken up and stored in the postganglionic noradrenergic nerve terminals similarly to norepinephrine, thereby providing an in vivo measure of sympathetic nerve terminal integrity.

**Figure 4:**
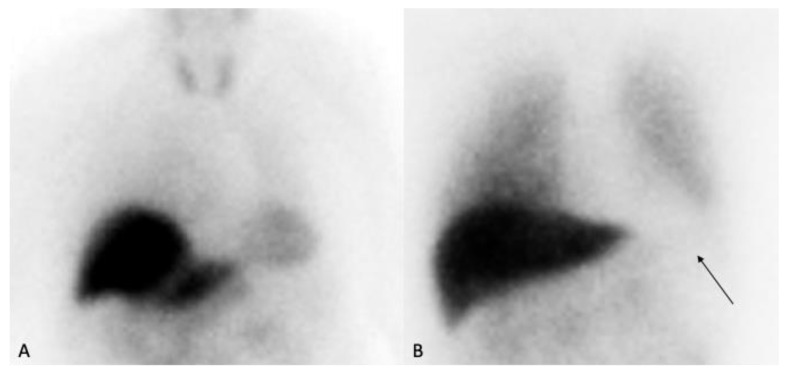
(**A**) Normal control showing physiological myocardial uptake. (**B**) Patient with markedly reduced cardiac uptake (arrow), consistent with abnormal sympathetic denervation.

In PD, numerous studies have documented a significant reduction in myocardial [^123I]MIBG uptake, evident both visually on planar images and quantitatively via heart-to-mediastinum (H/M) ratios (Figure 4).

Typically, healthy subjects exhibit increased H/M ratios over time from early to delayed images, whereas PD and DLB patients show an inverse pattern due to impaired tracer retention resulting from a reduced number of storage vesicles in damaged sympathetic terminals [94]. Recent advances in 3D tomographic imaging have further enhanced diagnostic accuracy by enabling detailed regional analysis of cardiac segments [95,96].

Cardiac [^123I]MIBG uptake is diminished in approximately 80–90% of patients with clinically probable PD [32,97,98,99,100], though lower rates are observed in early-stage PD. In contrast, atypical parkinsonian disorders such as PSP, CBD and MSA typically exhibit normal or near-normal cardiac [^123I]MIBG uptake [32]. Meta-analyses have provided more precise estimates of diagnostic performance. Orimo et al. and Treglia et al. reported pooled sensitivities of 83–88% and specificities of 85–86% for distinguishing PD from non-synucleinopathy parkinsonian syndromes, using delayed H/M ratio cut-offs primarily in the 0.93–1.8 range depending on camera/collimator systems and institutional calibrations [98,101]. Notably, the commonly adopted thresholds in these analyses were H/M < 1.7 for delayed images and H/M < 1.9 for early images, values that consistently separated PD/DLB from atypical parkinsonism across included cohorts.

Reduced cardiac [^123I]MIBG uptake is commonly observed in individuals with RBD, many of whom eventually develop an α-synucleinopathy [102,103]. A combined imaging approach using both MIBG and DAT SPECT has been shown to yield high sensitivity (95%) and specificity (91%) for differentiating dementia with Lewy bodies from Alzheimer’s disease (AD), wherein both cardiac and nigrostriatal dopaminergic innervation are typically preserved [104]. Although some studies have noted inverse correlations between cardiac [^123I]MIBG uptake and disease stage or motor symptom scores, the findings have not been uniformly consistent [101,105]. Additionally, orthostatic hypotension, common in treated PD, shows only a weak correlation with cardiac MIBG findings, especially in early disease stages [106,107,108,109].

## 6. Neural Connectivity, Cerebral Blood Flow, and Metabolism

PD induces widespread alterations in brain function, manifesting as changes in blood flow, oxygen and energy utilization, and interregional connectivity compared to healthy brains. The cerebral metabolic rate of glucose, an indirect marker of neuronal activity and synaptic integrity, is commonly quantified with the PET tracer [^18F] fluorodeoxyglucose ([^18F]FDG) [110]. Additionally, cerebral blood flow can be evaluated using SPECT tracers like 99mTc-hexamethylpropylene amine oxime ([^99mTc]Tc-HMPAO) and 99mTc-ethyl cysteinate dimer ([^99mTc]Tc-ECD), as well as the PET tracer [^15O]H_2_O [53]. Moreover, PET-based measurements of metabolic activity and blood flow can be translated into assessments of functional connectivity between different brain regions [110,111]. Such imaging investigations may be performed not only during resting conditions but also while subjects engage in tasks that activate brain regions implicated in PD [112,113,114].

### [^18F] FDG PET Imaging

The [^18F]FDG radiotracer provides a measure of cerebral glucose metabolism, closely reflecting neuronal activity and synaptic function [115]. Using Statistical Parametric Mapping (SPM), group comparisons between patients and controls have shown that PD typically displays a characteristic metabolic signature composed of relative hypermetabolism in the putamen, thalamus, cerebellum, pons, and sensorimotor cortex, together with hypometabolism in lateral frontal and parieto-occipital cortices [116]. Quantitatively, FDG-PET pattern analysis yields sensitivities of 80–90%, specificities of 85–92%, and AUC values of 0.88–0.94 for distinguishing PD from atypical or non-degenerative parkinsonism [117].

Although similar metabolic patterns have been reported in DLB, some studies have observed a more pronounced hypometabolism in the occipital cortex in DLB compared to controls [118,119,120]. In differential diagnosis against AD, occipital hypometabolism combined with sparing of the posterior cingulate (“cingulate island sign”) provides sensitivities of 83–92%, specificities of 80–90%, and AUC values in the 0.90–0.95 range [121].

To examine individual metabolic variability, Carli et al. applied an SPM-based single-subject approach comparing each [^18F]FDG PET scan from patients with iRBD to a large normative cohort [120]. This method achieved sensitivities of 78–85% and specificities of 82–90% for detecting abnormal metabolic patterns at the individual level, revealing heterogeneous phenotypes such as isolated occipitoparietal hypometabolism or combined occipital–cerebellar reductions. Some of these patterns were interpreted as suggestive of prodromal MSA.

Longitudinal studies in iRBD have demonstrated progressive metabolic changes, including increases in the putamen and decreases in the bilateral premotor cortex, supplementary motor area, and superior frontal gyrus [122,123]. Trajectory analysis discriminates future converters to PD or DLB from those progressing to MSA with classification accuracies of 75–85% and AUC values around 0.80–0.86, although findings involving the occipital cortex remain variable.

Dang-Vu et al. investigated regional cerebral blood flow with PET over three years in iRBD patients [124]. Hippocampal hyperperfusion predicted phenoconversion with sensitivities of 70–82%, specificities of 75–88%, and correlations with motor performance and color vision scores. However, perfusion metrics did not clearly differentiate converters to PD versus DLB.

## 7. Tau and Beta-Amyloid Imaging

Tau is a microtubule-associated protein essential for intracellular transport [125], while beta-amyloid is derived from the amyloid precursor protein (APP), which plays a role in neuronal growth regulation [126]. In AD, hyperphosphorylated tau and beta-amyloid fibrils are defining pathological features [127,128], whereas idiopathic PD is typically not marked by significant tau or beta-amyloid accumulation [129]. Nonetheless, postmortem studies indicate that patients with DLB and PD dementia may display beta-amyloid deposition in the striatum [130], and individuals with PSP exhibit tau aggregates in multiple brain regions [131]. Consequently, imaging beta-amyloid and tau aggregates becomes pertinent for distinguishing atypical parkinsonian syndromes.

Among the most widely used PET tracers for beta-amyloid imaging are [^11C] Pittsburgh compound B ([^11C]PIB), [^18F]florbetaben, [^18F]florbetapir, and [^18F]flutemetamol, with the latter three approved for AD diagnosis in both the US and Europe [127,131]. Amyloid PET has demonstrated sensitivities of 80–93%, specificities of 85–95%, and AUC values around 0.90–0.96 for distinguishing DLB from PD, largely driven by the higher cortical amyloid burden in DLB relative to PD [132]. Elevated uptake of [^11C] PIB has been reported in the cortices of DLB patients compared to those with PD [133], though the association between [^11C]PIB uptake and cognitive dysfunction in PD remains debated [134]. For tau imaging, the PET tracer [^18F]AV-1451 has recently received regulatory approval in the US [135]. Quantitative analyses show that tau-PET differentiates PSP from PD with sensitivities of 80–90%, specificities of 85–92%, and AUC values between 0.88 and 0.94, particularly when assessing binding in the globus pallidus, putamen, subthalamic nucleus, midbrain, and dentate nucleus [136]. Similar regional binding patterns—and comparable diagnostic performance, have been reported using the tau tracer [^18F]FDDNP [137].

Overall, beta-amyloid and tau PET imaging offers diagnostic accuracy in the moderate-to-high range for distinguishing PD, DLB, and PSP, and may become increasingly useful as a complementary tool for differential diagnosis in parkinsonian syndromes.

## 8. PET/MRI Systems in PD and Parkinsonism

Integrating nuclear imaging biomarkers, such as DAT or VMAT2 PET/SPECT, with MRI measures like neuromelanin imaging, quantitative susceptibility mapping (e.g., QSM), diffusion metrics, and resting-state fMRI through PET/MRI systems enables a multimodal assessment of dopaminergic loss, nigral integrity, iron accumulation, microstructural damage, and network dysfunction. Indeed, In the context of PD, hybrid scanners allow direct coupling of dopaminergic PET measures with MRI-derived markers such as neuromelanin signal loss, diffusion abnormalities, resting-state network disruption, and iron-sensitive metrics like QSM [138]. In particular when combined with nuclear imaging, QSM can enhance diagnostic specificity by simultaneously capturing functional dopaminergic loss (PET/SPECT) and structural iron-mediated neurodegeneration (QSM). Evidence from Langkammer et al. demonstrated that QSM-derived nigral iron metrics reliably distinguish patients with PD from controls and correlate with clinical motor impairment, supporting its potential as an early-stage or even prodromal biomarker [139].

Hence PET/MR fusion could help refine early diagnostic algorithms, enhance the detection of prodromal PD, and support mechanistic studies targeting the interplay between iron accumulation, dopaminergic vulnerability, and network-level dysfunction. Moreover, this combination captures complementary aspects of PD biology, improving diagnostic accuracy and differential diagnosis with atypical parkinsonian syndromes.

## 9. New Targets in PD Imaging

### 9.1. Alpha-Synuclein

The aggregation of α-Syn is considered the initiating event in PD pathogenesis, ultimately leading to neuronal loss and the spread of misfolded protein aggregates throughout the central and peripheral nervous systems. It is widely anticipated that developing effective molecular imaging agents for α-Syn will revolutionize PD research and diagnostics [133,140,141,142,143,144,145]. Such tracers could enable the early detection of pathological changes, monitor disease progression, and evaluate the impact of anti-PD therapies targeting α-Syn. Despite the recent efforts in developing a PET imaging probe for imaging α-Syn in the living human brain, no suitable radiotracers are currently available. The major obstacle to this achievement has been the absolute concentration of α-Syn inclusions, which are present at a much lower density of other misfolded proteins, such as Aβ and tau [141,142,143,144,145,146]. Consequently, a high affinity probe, in the subnanomolar range is required to successfully image α-Syn inclusions in the human brain. Selectivity of the ligand for the target is also crucial. In Parkinson’s disease high selectivity of the ligand for α-Syn is needed to overcome the higher and coexistent concentration of Aβ and tau [147]. Unfortunately, α-Syn, Aβ, tau fibrils, all share a common supersecondary structure: the cross β-sheet conformation, further complicating the development of selective α-Syn binding ligands [148]. In contrast to Aβ, α-Syn inclusions are found intracellularly, therefore a suitable α-Syn PET tracer must be able to cross the blood–brain barrier and the cell membranes [146]. Furthermore, α-Syn aggregates are subject to several post-translational modifications, such as methionine sulfoxidation, tyrosine nitration, serine phosphorylation, which affect the ultrastructural conformation of the aggregates and consequently the tracer binding [149,150]. The in vivo selectivity of α-Syn PET imaging agent is not easy to assess and needs an extensive pre-clinical characterization. In vitro validation includes the employment of radioligand binding assays using recombinant α-Syn fibrils and brain homogenates derived from patients to determine the maximum number of binding sites (B_max_) and the dissociation constant (K_d_) of the compound [151]. Notably, the structures of α-syn filaments extracted from both LBD and MSA brains differ from those formed in vitro using recombinant protein [142], therefore the employment of in vitro autoradiography in combination with neuropathological studies using human post-mortem brain tissue from patients with a confirmed diagnosis of neurodegenerative diseases is the most reliable tool for the pre-clinical evaluation.

Recent research efforts have focused on optimizing several classes of compounds to identify a PET tracer capable of targeting α-Syn [152]. In silico docking studies have indicated that candidate compounds bind to different subsets of sites on α-Syn fibrils [144,145,153]. For example, the phenothiazine derivative [^11C]SIL5, with an affinity of approximately 30 nM for α-Syn, demonstrated adequate brain penetration in rodents and primates, although its affinity is insufficient for human imaging [145,154]. Modifications to the indolinone scaffold produced [^18F]WC58a, with an improved affinity of 9 nM; however, its high lipophilicity limits the in vivo applicability [155]. Similarly, a chalcone derivative, IDP-4, with an affinity around 5 nM, exhibited low brain uptake and slow clearance in mice, likely due to its pronounced hydrophobicity [156]. Further refinement of the chalcone scaffold yielded more polar derivatives (e.g., compounds **11a** and **11b**) with α-Syn affinities in the range of 18.5 nM, which are now being used as lead structures for additional structure-activity relationship studies [153]. More promising results have been achieved with pyrazole derivatives, such as [^11C] anle253 and [^11C]MODAG-001. In vitro evaluations of these compounds have shown very high affinities for α-Syn fibrils (with IC_50 and K_d_ values of 1.6 nM and 0.6 nM, respectively), surpassing those of previous candidates. Notably, [^11C]MODAG-001 demonstrated a 30-fold preference for α-Syn over tau and beta-amyloid fibrils. PET imaging in rats confirmed that both [^11C]anle253 and [^11C]MODAG-001 exhibit favorable brain penetration and washout kinetics. Efforts to reduce radiometabolite formation by deuteration (resulting in (d_3)-[^11C]MODAG-001) have shown promising in vivo results, with the tracer accumulating at the site of recombinant α-Syn fibril inoculation in the striatum [144,145,157]. Nevertheless, challenges remain, as tritiated [^3H]MODAG-001 did not demonstrate specific binding to α-Syn in brain slices from DLB patients, possibly due to insufficient affinity under assay conditions, high nonspecific binding, or structural differences between in vitro and in vivo α-Syn aggregates [157].

Lastly, although α-Syn aggregation is a common hallmark of PD, DLB and MSA, emerging evidence suggests that future molecular imaging may differentiate these disorders by exploiting their distinct spatial topographies and aggregation kinetics. Indeed, in contrast to intraneuronal α-Syn inclusions in the form of Lewy bodies and Lewy neurites in PD and DLB [158], α-Syn inclusions in MSA occur predominantly in the form of glial cytoplasmic inclusions [159]. In principle, a sensitive and specific α-Syn PET tracer could reveal predominantly presynaptic nigrostriatal involvement in PD, cortical-limbic patterns in DLB, and prominent oligodendroglial pathology within striatum, pons, and cerebellum in MSA. Recent findings from cryo-EM analysis from human brain derived material reveal multiple ultrastructure of α-Syn filaments among α-synucleinopathies [160,161]. In this regard, selective α-syn PET tracers could be highly specific and bind to only one kind of α-Syn ultrastructural conformation, or be less specific, binding to all α-Syn conformations; therefore, a pathognomonic regional brain distribution of the tracer will help to identify distinct α-synucleinopathies.

While no α-Syn PET tracer is clinically validated yet, ongoing developments indicate that spatially resolved, quantitative α-Syn imaging could eventually support differential diagnosis across synucleinopathies.

### 9.2. Microglia

Microglia are specialized macrophage-like cells that constitute a key element of the brain’s immune system [162]. Upon activation, typically in response to injury or neurodegenerative processes, microglia undergo phenotypic changes and upregulate specific proteins. Notably, post-mortem analyses of PD brains have revealed elevated numbers of activated microglia in the substantia nigra. These activated cells express increased levels of the mitochondrial translocator protein (TSPO), which has thus been proposed as a potential biomarker for neuroinflammation [163,164]. The degree of neuroinflammation in PD has been explored using TSPO-targeted PET tracers. Initial studies employing the first-generation TSPO ligand (R)-[^11C]PK11195 reported heightened tracer uptake in various brain regions, including the midbrain, pons, and cortex, in PD patients relative to healthy controls, supporting the notion that neuroinflammation plays a role in PD pathology [165]. One investigation even demonstrated a positive correlation between (R)-[^11C]PK11195 uptake and PD motor severity [166]. However, subsequent studies utilizing second-generation TSPO tracers such as [^11C]PBR28 and [^18F]FEPPA did not replicate these group differences between PD patients and controls [167,168]. These discrepancies highlight several unresolved translational barriers, including the well-known influence of the rs6971 TSPO polymorphism on ligand-binding affinity, variability in cellular specificity, and methodological challenges in achieving robust quantification across centers [169]. Although newer “third-generation” tracers aim to reduce genotype sensitivity and improve pharmacokinetic properties, large-scale validation studies are still lacking.

Furthermore, complementary measures, including CSF biomarkers (e.g., cytokines, soluble TREM2) and TSPO-independent PET tracers targeting alternative pathways such as P2X7 or COX-1, can provide additional insight into neuroinflammatory processes [170]. Consequently, while TSPO PET offers valuable information, careful consideration of genetic variability and integration with complementary approaches is essential to reliably interpret microglial imaging in PD [171]. At present, ongoing human studies are beginning to address these limitations, but a reliable transition to routine clinical application will require standardized protocols, multicenter reproducibility, and clearer demonstration of disease-specific signals.

## 10. What’s New Since 2020: Multicenter Imaging, and SV2A/Synaptic Density

Recently, benzothiazole molecules have been investigated as promising candidates for the development of selective PET tracers for α-Syn aggregates. Endo and colleagues have reported pre-clinical and preliminary clinical findings for their tracer, [^18F]-C05-05 [172]. The compound demonstrated high reactivity for visualizing α-Syn aggregates in human post-mortem brain tissue and exhibited favorable properties as an in vivo imaging agent, successfully detecting α-Syn inclusions in living murine and non-human primate (NHP) models through optical and PET imaging. However, the authors also found evidence for binding to amyloid-beta and tau, indicating that the compound may not be specific to α-Syn pathology. However, PET imaging with [^18F] C05-05 revealed intensified signals in the midbrains of PD and DLB patients compared to healthy controls, providing the first successful visualization of Lewy-type α-Syn pathologies in humans [172]. In parallel to the development of C05-05, a partnership known as the Synuclein PET Alliance (SPAL), lead to the development of a C05-05 analog, called SPAL-T-06 [173]. Preliminary in vitro studies conducted on post-mortem human brain tissue from MSA and control cases showed promising results. Clinical studies using [^18F]SPALT-06 in MSA and control cases indicated uptake in the basal ganglia of individuals with MSA-P and in the pons and cerebellar structures of MSA-C patients [173]. No uptake was found in PD and DLB and minimal binding in control individuals. Another benzothiazole derivative, that has emerged as a promising lead compound for imaging α-Syn inclusions is F0502B, which selectively binds to α-Syn but not to Aβ and tau fibrils [174]. Xiang et al. found that [^18F]F0502B selectively labels α-Syn aggregates in mouse, monkey PD models and human post-mortem brain tissue and the in vivo PK studies demonstrated that F0502B possesses favorable brain permeability and is washed out of the normal brain swiftly [174]. Further studies using this compound are still ongoing. In 2022, Capotosti et al. identified through AC Immune’s proprietary Morphomer library [^18F]ACI-12589 [175]. Binding studies revealed a Kd for familial PD and MSA of 17nm and 28nM, respectively. In vitro autoradiography confirmed target engagement to α-Syn inclusions in a wide range of α-synucleinopathies and limited interaction with Aβ, tau and TDP-43 aggregates [143]. Clinical studies of 42 participants revealed that [^18F]ACI-12589 demonstrated high stability and strong PET signals in cerebellar regions, especially in cerebellar ataxia-dominant MSA, but not in participant with PD and DLB, making it a potential diagnostic tool for MSA disease [176]. Benzamide derivatives has also been evaluated as potential PET imaging probes for α-Syn. Among these ligands, two main compounds, [^11C]HY2-15 and [^11C]M503, have been identified with PET (CW_2_IP_2_) [143,177]. In vitro autoradiography studies revealed that [^3H]HY2-15 can detect α-Syn aggregates in MSA post-mortem human brain tissue but not in PD, while [^3H]M503 is able to detect α-Syn inclusions found in PD, PD dementia and DLB brain tissue but not in MSA [176,177]. These tracers are currently being explored in clinical PET programs. Although the development of a PET tracer that specifically targets α-Syn aggregates is challenging, substantial progress has been made, enabling better understanding of this complex task.

Moreover, multicenter imaging initiatives have expanded, promoting standardized evaluation of synucleinopathies across multiple sites. High-throughput post-mortem studies of synaptic markers, including SV2A and synaptophysin, have revealed region-specific synapse loss that correlates with α-Syn burden, neurofilament light accumulation, and cognitive decline in PD, PD dementia, and DLB [178]. Such findings support the establishment of multicenter consortia to harmonize imaging protocols and validate synaptic biomarkers on a large scale.

Finally, SV2A PET imaging, as a marker of synaptic density, has demonstrated both clinical and methodological advancements. Deep-learning models have enabled the generation of synthetic SV2A PET images from structural MRI, achieving high fidelity relative to real PET scans [179,180]. In vivo studies have identified early synaptic loss in cognitively unimpaired high-risk individuals, suggesting that synaptic alterations are detectable prior to overt clinical symptoms [181]. Post-mortem analyses corroborate these observations, confirming region-specific SV2A reductions that correspond to α-Syn pathology and clinical severity [181].

Collectively, these developments illustrate a rapidly maturing field. α-Syn PET tracers are transitioning toward clinical testing, multicenter imaging collaborations are reinforcing the link between neuropathology and imaging, and SV2A PET is consolidating as a quantitative biomarker of synaptic integrity. This convergence offers unprecedented opportunities to monitor disease progression and evaluate emerging disease-modifying therapies.

## 11. Conclusions

In conclusion, molecular imaging is emerging as an indispensable tool not only for refining the clinical diagnosis of PD but also for probing its underlying pathophysiology beyond the limitations of symptom-based approaches. PET and SPECT techniques, through the use of highly specific radiotracers, have already transformed our ability to assess dopaminergic dysfunction, cardiac sympathetic denervation, and cerebral metabolism. However, the most transformative potential of molecular imaging lies in its capacity to detect neuropathological changes at the preclinical stage, when interventions are more likely to be effective. Looking ahead, the development of novel radiotracers targeting the primary pathogenic drivers of PD, such as α-Syn aggregation, neuroinflammation, mitochondrial dysfunction, and oxidative stress, is of paramount importance. Imaging α-Syn in vivo, in particular, represents a critical advancement for the identification of early biomarkers, patient stratification, and monitoring the efficacy of disease-modifying therapies, which remain an unmet clinical need.

## Figures and Tables

**Table 1:** This table summarizes PET and SPECT imaging modalities used in current clinical practice for PD and atypical parkinsonian syndromes. Principal features for each disease are described.

Imaging Modality	Idiopathic Parkinson’s Disease (PD)	Multiple System Atrophy (MSA)	Progressive Supranuclear Palsy (PSP)	Corticobasal Degeneration (CBD)	Dementia with Lewy Bodies (DLB)
DAT SPECT/PET	Asymmetric posterolateral putaminal reduction;	Marked, symmetric putaminal loss;	Marked caudate + putamen loss; less asymmetry;	Asymmetric contralateral reduction;	Reduced uptake similar to PD more symmetric;
[^18F]FDOPA PET	Decreased putaminal uptake;	Severe, symmetric putaminal + caudate reduction;	Widespread striatal loss;	Asymmetric reductions;	Reduced, but less specific for DLB;
Cardiac MIBG Scintigraphy	Markedly reduced H/M ratio;	Typically normal or mildly reduced;	Normal;	Normal;	Markedly reduced, similar to PD;
FDG PET	Hypermetabolism in pallidothalamic + cerebellar networks,	Cerebello-pontine/putaminal hypometabolism;	Frontal + midbrain hypometabolism;	Asymmetric frontoparietal hypometabolism;	Occipital hypometabolism characteristic;

**Table 2:** Emerging PET and SPECT radiotracers under investigation for PD. These agents target diverse molecular mechanisms such as α-Syn aggregation, neuroinflammation (TPSO), and synaptic density, but are not yet approved for clinical use (C = clinical; R = research; Pre-C = pre-clinical).

Target	Imaging Modality	Radiotracer	Clinical Role	Stage	Developmental Stage
VMAT2	PET	[^11C]DTBZ [^18F]AV-133;	Presynaptic density	C/R	FDA/EMA-approved tracers in use; others in clinical validation;
Post-synaptic D2/3 receptors	PET/SPECT	[^11C]raclopride [^123I]IBZM [^18F]fallypride;	Differentiation PD vs. Atypical Parkinsonism,	C/R	Mostly established tracers; high-affinity D3-selective agents under development;
Tau protein	PET	[^18F]AV1451, [^18F]FDDNP	Differentiation of PSP, CBD, DLB	R	Second-generation tau tracers in early clinical testing;
TSPO	PET	[^11C]PK11195, [^11C]PBR28 [^18F]FEPPA	Assessment of microglial activation	R	Several second-generation TSPO ligands in clinical research; no approved clinical use;
α-Syn	PET	[^11C]MODAG-001, [^11C]anle253, IDP-4;	Imaging of α-Syn aggregates	Pre-C	All α-Syn tracers remain preclinical; first-in-human studies ongoing or planned;

## Data Availability

No new data were created or analyzed in this study.
